# Increased Macrophage-like Cell Density in Retinal Vein Occlusion as Characterized by en Face Optical Coherence Tomography

**DOI:** 10.3390/jcm11195636

**Published:** 2022-09-24

**Authors:** Wenyu Wang, Gongpeng Sun, Lu He, Changzheng Chen

**Affiliations:** 1 Department of Ophthalmology, Renmin Hospital of Wuhan University, 238 Jiefang Road, Wuhan 430060, China; 2 Physical Examination Center, Renmin Hospital of Wuhan University, Wuhan 430060, China

**Keywords:** OCT, retina, retinal vein occlusion (RVO), macrophage-like cells (MLCs)

## Abstract

Objectives: to quantitatively analyze macrophage-like cells (MLCs) at the vitreoretinal interface in retinal vein occlusion (RVO) using swept-source optical coherence tomography angiography (SS-OCTA) and en face optical coherence tomography (OCT). Methods: The study included 72 RVO patients, with 43 acute patients and 29 chronic patients. For a normal control, 64 fellow eyes were included. MLCs were visualized in a 5 μm en face OCT slab above the vitreoretinal interface centered on the fovea. After semi-automatic binarization and quantification, we evaluated the MLC count and density among groups. We also investigated the MLC density and distribution relative to retinal edema. Results: Morphological changes and congregation of MLCs appeared in RVO eyes. The MLC density of both the acute and chronic groups was significantly higher than that of the control eyes (*p* < 0.001). In the acute group, the MLC density of the edematous region was lower than both the non-edematous region (*p* < 0.001) and the whole image (*p* < 0.01). The MLC density in acute eyes was negatively correlated to central fovea thickness (CFT) (r = −0.352, *p* < 0.05). The MLC density in chronic eyes was positively correlated to CFT and mean retina thickness (MRT) (r = 0.406, *p* < 0.05; r = 0.412, *p* < 0.05, respectively). Conclusions: SS-OCTA is a viable and simple method for the characterization of MLCs at the vitreoretinal interface. A significant increase in the MLC density in both acute and chronic eyes implicates the activation and recruitment of MLCs in RVO and that the MLC density and distribution can be affected by retinal edema.

## 1. Introduction

Retinal vein occlusion (RVO), including both the branch and the central type, is the second most common cause of vision loss due to retinal vascular diseases [1,2]. Both central retinal vein obstruction (CRVO) and branch retinal vein occlusion (BRVO) may lead to focal retinal atrophy of the inner retinal layers, especially in retinal ganglion cells (RGCs) and the retinal nerve fiber layer [3]. There is much evidence showing that an inflammatory response is involved in the pathological process of RVO and contributes to the associated retinal atrophy. A large quantity of research suggests that different kinds of cytokines and chemokines are elevated in vitreous samples from patients and animal models [4,5,6,7]. Additionally, an increase in inflammatory factors is associated with retinal edema [7,8]. Previous research has shown that tissue ischemia and the breakdown of the inner blood–retina barrier results in the activation of retinal microglia and the recruitment of blood-derived monocytes into hypoxic areas in experimental RVO [6,9,10].

Microglia in the retina are immune cells that originate from the monocyte–macrophage system and play a role in the immune response [11,12]. Macrophages in the vitreous humor are known as hyalocytes, parts of which are located above internal limiting membranes (ILM) [13,14,15]. Physiologically, they help to keep the transparency of the vitreous humor [16]. In previous animal studies, both microglia and hyalocytes were found to be involved in retinal pathology [17] and played an important part in several retinal diseases such as glaucoma [18], diabetic retinopathy (DR) [19], uveitis [20], idiopathic epiretinal membrane [21], and age-related macular degeneration [22]. However, most of the previous research has been conducted on animal models, using staining and confocal microscopy to identify these cells. Recently, some studies have reported that macrophages located at the ILM were successfully and consistently observed in live human retinas using adaptive optics with optical coherence tomography (AO-OCT) as well as clinical-used optical coherence tomography (OCT) [23,24]. These studies also described the distribution and dynamics of the cells [24]. Furthermore, another group observed an increase in macrophage-like cells (MLCs) at the vitreoretinal interface [25]. The above studies fully demonstrate the viability of the patients’ MLCs in the clinical setting.

Based on RVO studies using animal models and the little available research on human retina specimens, we presumed that macrophages were involved in the pathological process of RVO. We collected new-onset and treatment-naïve RVO patients as well as long-course treated RVO patients, aiming to observe changes in the quantity, distribution characteristics, and morphology of MLCs through clinical swept source OCT (SS-OCT) and SS-OCT angiography (SS-OCTA).

## 2. Materials and Methods

### 2.1. Subjects 

This cross-sectional study included patients with RVO seen between September 2021 and February 2022 at the Eye Center of the Renmin Hospital of Wuhan University in Wuhan, China. The study was approved by the Institutional Review Board of the Renmin Hospital of Wuhan University (WDRY2021-k162) and conducted in accordance with the tenets of the Declaration of Helsinki. Informed consent was obtained from all participants. All subjects were diagnosed based on fundus fluorescein angiography (FFA), color fundus photography (CFP), or ultra-wide field fundus fluorescein angiography (UWFA) images. CRVO was characterized on CFP, FFA, or UWFA by disc oedema, increased dilatation and tortuosity of all retinal veins, widespread deep and superficial retinal hemorrhages, cotton wool spots, and retinal edema and capillary non-perfusion in all four quadrants of the retina. BRVO was defined as similar features to CRVO, except that they were confined to that portion of the fundus drained by the affected vein [26]. In the BRVO group, only patients with macular involved were included. Patients with a disease course ≤ 90 days were defined as acute RVO patients. Both eyes of all patients underwent comprehensive ophthalmologic examinations, including visual acuity, fundus fluorescein angiography, color fundus photography, and slit lamp examination. The medical history in ophthalmology and treatment of participants were also recorded. Exclusion criteria included: (1) prior ocular trauma or surgical procedures such as pars plana vitrectomy; (2) concomitant with other retinal diseases (glaucoma, diabetic retinopathy, uveitis, neovascular age-related macular degeneration, pathologic myopia, etc.); (3) refractive media opacity or poor fixation affecting imaging quality; (4) eyes with large area of epiretinal membrane (ERM) affecting image analysis; (5) subjects with refractive error > 3.0 diopters (D) or <−6.0 D.

### 2.2. OCTA Image Acquisition

OCTA images were acquired using a commercial SS-OCTA instrument (VG100, SVision Imaging, Ltd., Luoyang, China) equipped with a 1050-nm-wavelength laser. OCTA was performed using a raster scan protocol of 512 (horizontal) × 512 (vertical) that covered an area of 6 mm × 6 mm centered on the fovea. To reduce artifacts and to increase the credibility of the results, we repeated images (range, 3–5 repeats) at the same location in the retina. The study only included the OCTA images with quality scores greater than or equal to 7. We segmented the MLC layer at the ILM interface using a 5-μm OCT slab located from 5 to10 μm above the ILM; this way was the clearest location for visualization of MLCs. The segmentation lines were adjusted manually if needed.

### 2.3. Image Processing 

We first derived the en face OCTA images of the MLC layer from the software and then imported the images into ImageJ software for cropping and preprocessing. Since the en face macrophage layer was obtained through screenshots, we first clipped outside of the MLC images. Second, the square (radius = 5), horizontal line (radius = 10), vertical line (radius = 10), line 45 degrees (radius = 10), and line 135 degrees (radius = 10) in the white top hat algorithm were successively applied to enhance MLC imaging and to remove some artifacts. Larger artifacts were further removed through the subtract background (rolling = 10). Finally, the image in the previous step was copied and Gaussian blur was applied (sigma = 10). After subtracting the Gaussian blur from the image in the previous step, we obtained the preprocessed macrophage images. To extract MLCs from the images, several images with clear macrophage structure were selected and trainable WeKa segmentation was used to mark MLCs and the background. After that, an ideal training model was obtained, which was then used to extract MLCs from the preprocessed image. The resulting image was first converted to an 8-bit image and then binarized. The analyze particles tool was used to analyze the number, area, and percentage of MLCs.

### 2.4. Retinal Parameters Acquisition

**Figure 1:**
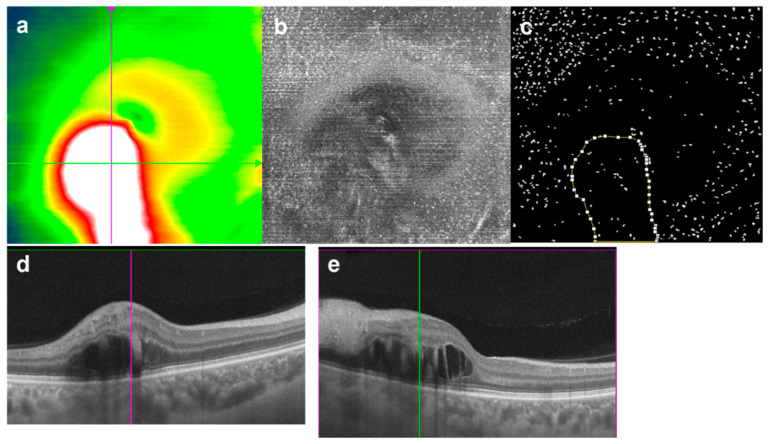

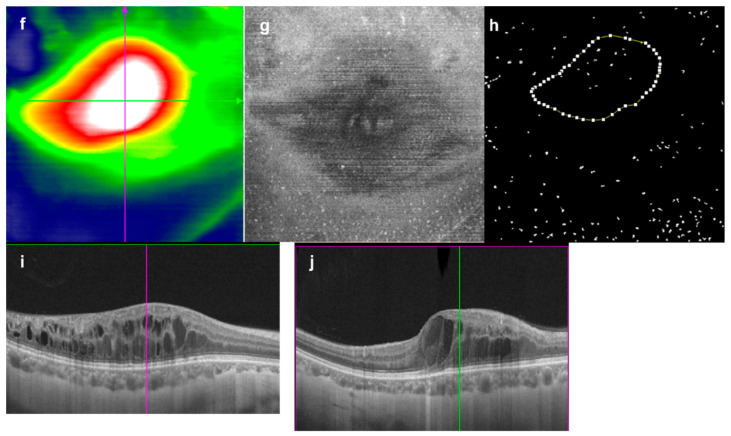
MLC density decreased significantly in the edematous region (**a**–**e**) and (**g**–**j**) from two different acute patients with retinal edema. (**a**,**f**) Retina thickness map. (**b**,**g**) En face 5 μm OCT slabs above ILM with hyperreflective dots representing MLCs. (**c**,**h**) Binarized image with the edema region boundaries outlined. The edema region marked by red edge in (**a**,**f**). (**d**,**e**,**i**,**j**) OCT B-scans showing the retinal edema. The colored lines in (**d**,**e**,**i**,**j**) corresponding to the lines with same color in (**a**,**f**), respectively.

Edema regions were manually divided according to the retinal thickness distribution map created by the built-in software, version 1.36.4 (examples shown in Figure 1a,f). The red and white areas in Figure 1 were considered as edema regions, representing regions with retinal thickness greater than 440 μm. The edema region was measured semi-automatically using ImageJ software. The vessel density (VD) of the deep capillary complex (DCP), superficial capillary complex (SCP), mean retina thickness (MRT), and central fovea thickness (CFT) were also automatically measured by the built-in software of the SS-OCTA instrument.

### 2.5. Statistics

We used SPSS Statistics (version: 26.0; IBM Corp., Armonk, NY, USA). Continuous variables coincided with normal distribution are presented as the mean ± standard deviation. Continuous variables not coincided with normal distribution are presented as the median (interquartile range). After using the Shapiro–Wilk test to test the normality of the data and Levene’s test for the homogeneity test of variance, we performed independent samples Mann–Whitney U test to compare the OCTA parameters and the MLC parameters between RVO eyes and the control group. The Friedman test was used to compare the MLC parameters in the edematous region, non-edematous region, and the whole image. Significance values have been adjusted by Bonferroni correction. Based on the data distribution type, the Pearson correlation coefficient or the Spearman correlation coefficient was used to verify the correlation between the MLC count and the OCTA parameters as well as the correlation between RVO eyes and fellow eyes. Differences were considered statistically significant at *p* < 0.05.

## 3. Results

Seventy-two eyes of seventy-two patients with RVO were included in the study. Among them, BRVO patients accounted for 61.1% (44/72). Sixty-four patients’ fellow eyes that met the inclusion criteria were included as the control group. Demographic characteristics were shown in Table 1. Neither hemicentral nor hemispheric RVO eyes were included in the study. Time from disease onset to OCTA acquisition ranged from 5 days to 3 years (median time, 65.5 days). The acute group (n = 43, 60.0%) only included treatment-naïve patients. In the chronic group, there were just seven untreated patients, treatment for the other twenty-two patients included retinal photocoagulation, intravitreal injection of anti-VEGF drugs (including Ranibalumab, Aflibercept, and Conbercept), and intravitreal administration of steroids. Eight individuals (27.6%) received retinal photocoagulation (grid pattern), twenty patients received intravitreal injection of anti-VEGF drugs (69.0%; injection times range from 1 to 11; median, 3), and eight patients received intravitreal injection of steroids (28.6%; injection times range from 1 to 4; median, 2). 

**Figure 2:**
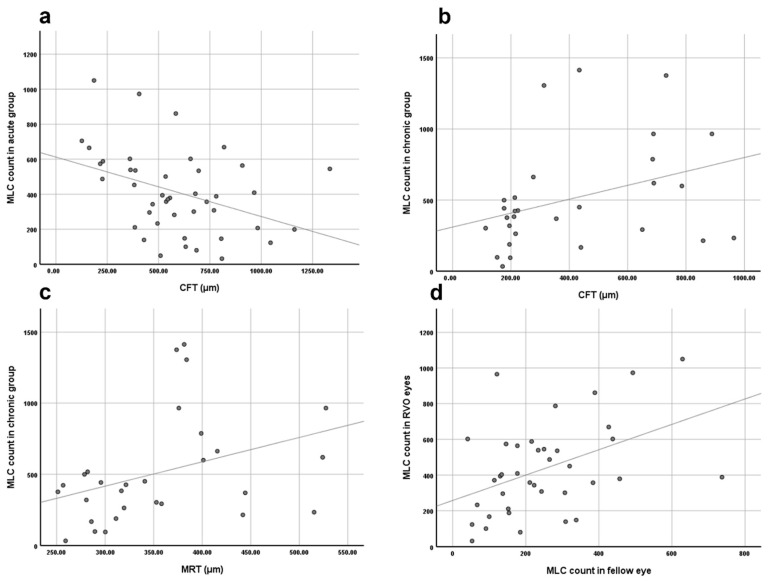
Results of correlation analysis. (**a**) The negative correlation between MLC count and central fovea thickness (CFT) in acute RVO eyes. (**b**) The positive correlation between MLC count and CFT in chronic RVO eyes. (**c**) The positive correlation between MLC count and mean retina thickness in chronic RVO eyes. (**d**) The positive correlation between MLC count in RVO eyes and in the fellow eyes.

**Figure 3:**
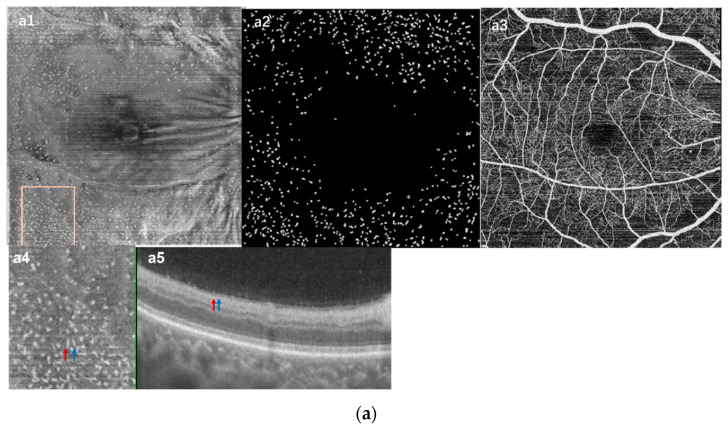

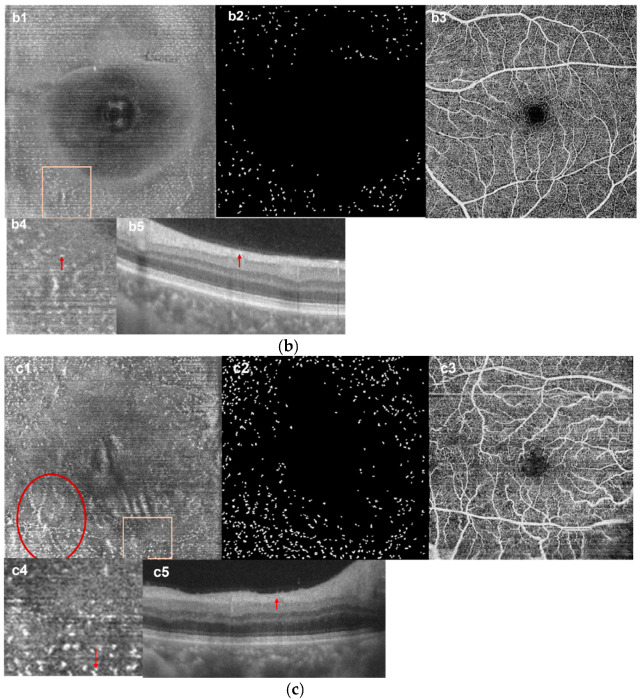

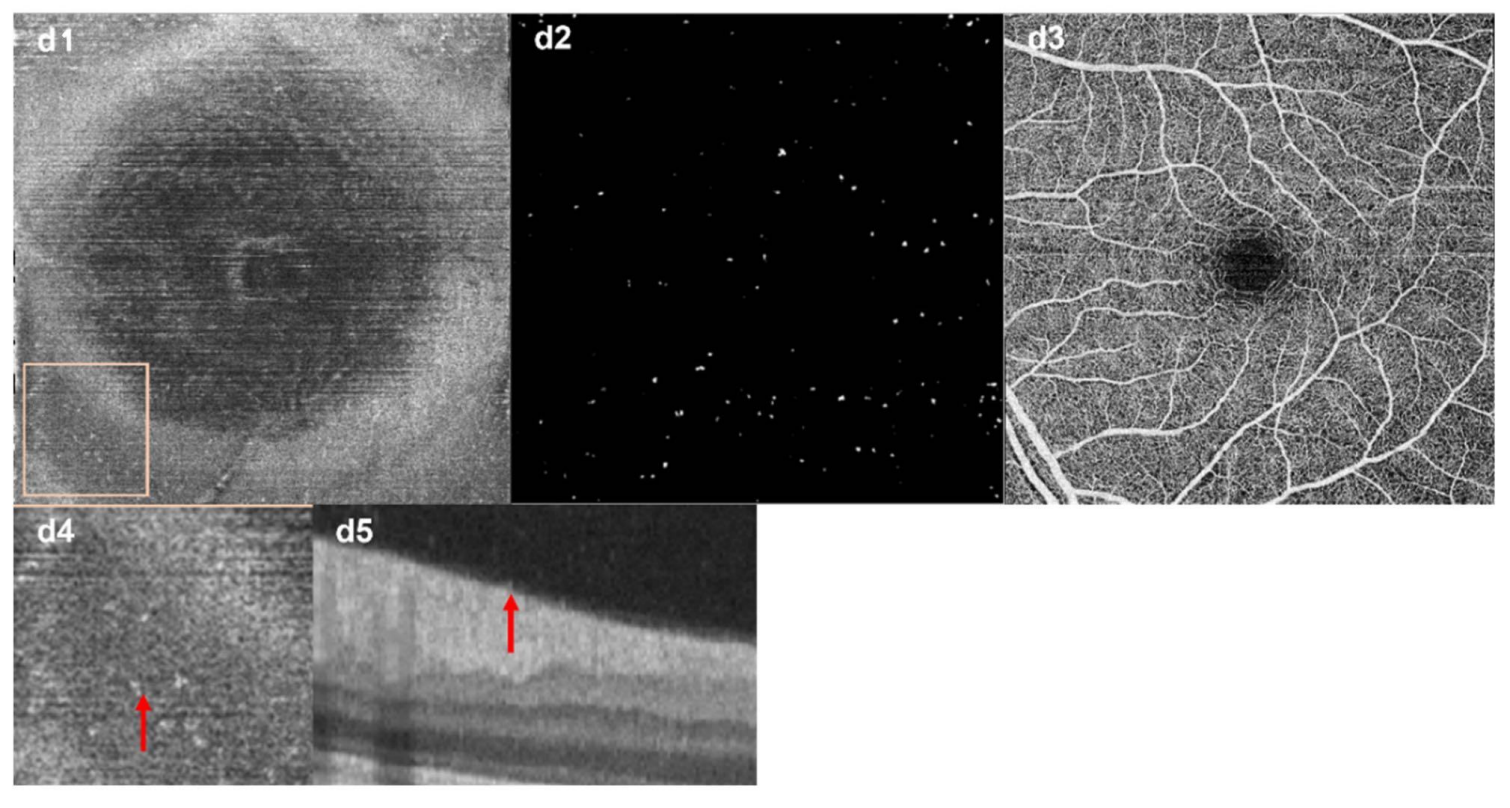

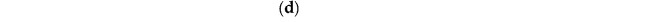
The density of MLCs increased in RVO eyes. (**a**) images from an acute CRVO patient’s affected eye. (**a1**) En face OCT slab of MLCs. (**a2**) Binarized image of (**a1**). (**a3**) OCTA images showing non-perfused regions. (**a4**) Magnified image from the pane in (**a1**). (**a5**) OCT B-scan images. MLCs on B-scan appeared as little embossment (red and blue arrows corresponding to the same color arrows in (**a4**)). (**b**) Images from the fellow eye of (**a**). (**b1**) En face OCT slab of MLCs. (**b2**) Binarized image of (**b1**). (**b3**) OCTA images. (**b4**) Magnified image from the pane in (**b1**). (**b5**) OCT B-scan images. Although MLCs of the normal eye on B-scan also appeared with little embossment (red arrow), it was not as obvious as in the RVO eye. (**c**) Images from another acute CRVO eye. (**c1**) En face OCT slab of MLCs, some of MLCs distributed along the blood vessels (red circle). (**c2**) Binarized image of (**c1**). (**c3**) OCTA images. (**c4**) Magnified image from the pane in (**c1**). (**c5**) OCT B-scan images (the red arrows of (**c4**,**c5**) correspond). (**d**) Images from the fellow eye of (**c**). (**d1**) En face OCT slab of MLCs. (**d2**) Binarized image of (**d1**). (**d3**) OCTA images. (**d4**) Magnified image from the pane in (**d1**). (**d5**) OCT B-scan images (the red arrows of (**d4**,**d5**) correspond).

The MLC parameters and OCTA parameters are summarized in Table 2. The MLC count and density showed a large variation between individuals, but those values for RVO eyes compared with fellow eyes showed a positive correlation (n = 64, r = 0.396, *p* = 0.014) (Figure 2d). Compared with fellow eyes, the MLC count and density increased significantly in RVO eyes (391.00 (352) vs. 220.50 (220), *p* < 0.001 and 11.61 (10.44) cells/mm^2^ vs. 6.55 (6.53) cells/mm^2^, *p* < 0.001) (Figure 3a vs. Figure 3b). No statistical difference was found in MLC count between CRVO and BRVO (454.50 (367) vs. 377.50 (345), *p* = 0.15). The MRT of RVO eyes was also greater than in the control eyes (388.05 (140.21) vs. 291.52 (16.56), *p* < 0.001). Additionally, vessel density, especially in deep vascular complexes (DCP), was greatly reduced in RVO eyes. Statistical analysis of the acute group and the chronic group found that the MRT and CFT in the acute group (time from onset ≤ 90 days) were significantly thicker than those of the chronic group (time from onset > 90 days) (422.40 (126.69) vs. 340.82 (112.48), *p* < 0.001; 555.31 (382.93) vs. 434.25 (493.4), *p* < 0.01, respectively). Although the acute group exhibited less MLCs than the chronic group, there was no statistical difference in the MLC count and density between the two groups (388.00 (353) vs. 422.00 (393), and 11.53 (10.37) cells/mm^2^ vs. 12.53 (11.66) cells/mm^2^, *p* = 0.22). In the chronic group, there was no significant difference in the MLC density, CFT, and MRT between the untreated and treated eyes. Moreover, no statistical significance was found between the subgroups divided according to types of treatment. 

**Figure 4:**
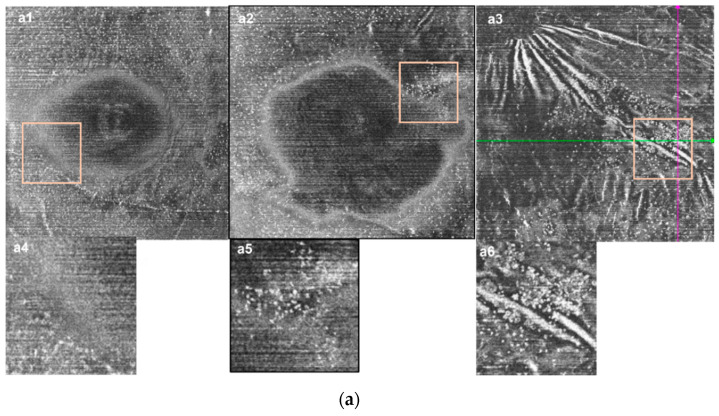

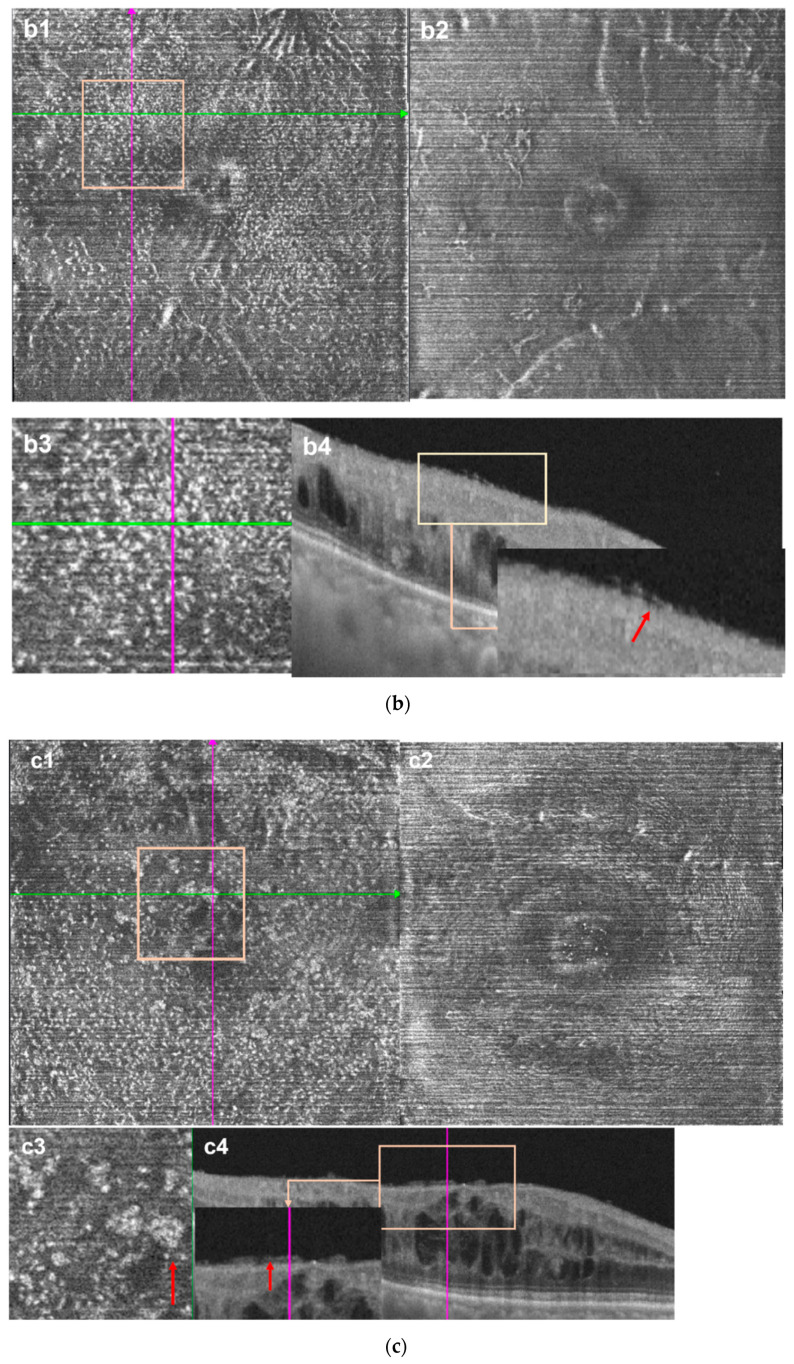
Distribution and morphology characteristic of MLCs. (**a**) The distribution and morphology of MLCs were different between normal, acute BRVO, and chronic BRVO eyes. (**a1**,**a4**) En face OCT image and the magnified image from the normal eye. The distribution of MLCs was relatively even. (**a2**,**a5**) En face OCT image and the magnified image from the acute RVO eye. Some MLCs congregated and the signal of MLCs was enhanced. (**a3**,**a6**) En face OCT image and the magnified image from the chronic RVO eye. MLCs were larger. Highly reflective clumpy structures (red arrow) and retinal folds (yellow arrow) are shown. (**b**,**c**) Images from chronic RVO eyes. (**b1**–**b4**) Images from a chronic BRVO patient with long-lasting retinal edema for more than 2 years. (**b1**) En face OCT slab from the affected eye. (**b2**) The en face OCT slab from the fellow eye, MLCs were barely visible. (**b3**) Magnified partial image from (**b1**). MLCs were larger and congregated. (**b4**) OCT B-scan and the magnified image on the green line in (**b1**). Retinal edema was shown. Connections between the MLCs (red arrow) are visible. (**c1**–**c4**) Images from a chronic BRVO patients with retinal edema. Compared with the fellow eye, the affected eye presented significantly increased MLCs with a clump-like structure. (**c1**) En face OCT slab from the affected eye. (**c2**) En face OCT slab from the fellow eye. (**c3**) Magnified partial image from (**c1**), hyperreflective clump-like structures are shown (red arrow). (**c4**) OCT B-scan and the magnified image on the green line in (**c1**), hyperreflective clump-like structures on B-scan are shown (red arrow). The purple line corresponded to the line of same color in (**c1**).

In addition to the increase in MLC numbers, we also observed morphological changes, including the increased size and signal enhancement in acute RVO eyes (Figure 3(a4,c4) vs. Figure 3(b4,d4)). Interestingly, the morphology of MLCs in chronic eyes was more remarkable. Comparing the acute group with the chronic group, we found that in some of the chronic RVO eyes (6/29), the size and number of MLCs increases greatly (examples shown in Figure 4). Furthermore, these abnormally larger cells gathered in clusters and accumulated in the macula, which was different from the normal eyes and the acute RVO eyes. On the OCT B-scans, connections appeared between the aggregated cells, which was never found in the acute eyes (Figure 4). The average disease course of the six patients was 322.7 days. All these eyes had retinal edema and underwent at least two rounds of intravitreal injection of anti-VEGF drugs and at least one round of intravitreal steroids.

**Figure 5:**
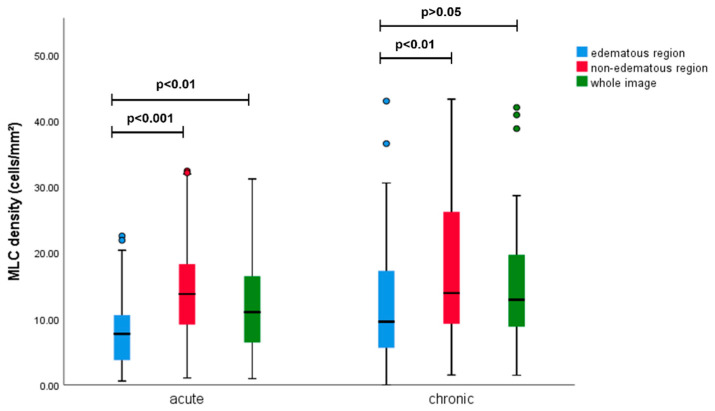
Comparison of MLC density between edematous area, non-edematous area, and whole image in the acute and chronic groups. In the acute group, MLC density reduced significantly in the edematous region compared with the non-edematous region and the whole image. In the chronic group, the MLC density reduced significantly in the edematous region compared with the non-edematous region. There was no significant difference in MLC density between the edematous region and the whole image in the chronic group.

We also investigated the distribution characteristics of MLCs. In the normal eyes, most MLCs were distributed evenly in the periphery of the en face OCT image and only a few cells were found within 3 mm of the fovea (Figure 3(b1,d1)). The MLCs in RVO eyes were found to congregate and move closer to the fovea (Figure 3a and Figure 4(a2,a3)). Distribution of MLCs along the blood vessels was more common in RVO eyes compared with the fellow eye (Figure 3(c1) vs. Figure 3(d1)). We further studied whether the location of the MLCs was affected by the edematous region by comparing MLC density in edematous regions with MLCs in non-edematous regions. Among seventy-two RVO eyes, sixty-three eyes had edematous regions that could be divided. The statistical results of these RVO eyes are shown in Table 3 and Figure 2. Compared with the non-edematous region and the whole image, the MLC density decreased significantly in the edematous area (7.91 (10.97) cells/mm^2^ vs. 13.85 (12.21) cells/mm^2^, adjusted *p* < 0.001; 7.91 (10.97) cells/mm^2^ vs. 11.70 (10.96) cells/mm^2^, adjusted *p* < 0.01, respectively). The difference of the distribution between edematous and non-edematous regions was more significant at the acute stage. In the chronic eyes, MLC density in the edematous region was slightly larger than in the acute eyes. Moreover, there was no statistical difference in MLC density between the edematous region and the whole image (Figure 5). Among acute patients, the edematous region area in the CRVO group was larger than the BRVO group (14.86 (19.27) mm^2^ vs. 10.21 (11.83) mm^2^, *p* = 0.045). However, the RVO type seemed to have no influence on MLC distribution. MLC density in the edematous region of the acute CRVO patients was similar to the acute BRVO patients (CRVO vs. BRVO, 6.50 (9.92) cells/mm^2^ vs. 8.31 (9.20) cells/mm^2^, *p* = 0.4). The same situation could also be found in the chronic patients (CRVO vs. BRVO, 11.33 (16.28) cells/mm^2^ vs. 7.45 (9.64) cells/mm^2^, *p* = 0.65).

To further understand the relationship between the retinal edema and the MLC count, we conducted a correlation study. Correlation analysis showed that in the acute group, the MLC count negatively correlated to CFT (n = 43, r = −0.352, *p* < 0.05). However, the MLC count in the chronic group positively correlated to CFT and MRT (n = 29; r = 0.406, *p* < 0.05; r = 0.412, *p* < 0.05, respectively). Moreover, there was no significant correlation between the MLC count and VD in either DCP or SCP. 

## 4. Discussion

In the current clinical study, we used an en face OCT image to investigate the density and morphological changes of MLCs located in the retinal vitreous interface in RVO eyes. The increasing number of MLCs and the morphological changes on the en face OCT indicated that these cells may be involved in the pathophysiology of RVO. We also found that some MLCs distributed along the blood vessels, which is consistent with a previous study in DR patients [25].

A substantial amount of evidence has shown that the inflammatory response is involved in the pathological process of RVO and contributes to retinal atrophy. The upregulation of inflammatory factors observed in the vitreous fluid also supports this view [27,28]. A previous study found the activation of microglia and the recruitment of macrophages from the systemic circulation in RVO experimental models [6]. Hyalocytes located above the inner surface of the retina are also considered to derive from the monocyte/macrophage lineage [14]. Furthermore, it has been suggested that hyalocytes might be one of the cellular sources of VEGF in DR and exudative age-related macular degeneration [28]. Although it is hard to study the origin and the function of these MLCs in our study using the clinical OCT device, the current study follows the previous experimental conclusions that the inflammatory response takes part in the pathological process of RVO in both acute and chronic stages. Specially, there are abnormally large MLCs gathering in clusters in one-fifth of the chronic RVO eyes, a finding not observed in either the acute RVO eyes or the normal eyes. Connections appeared between the aggregated cells on B-scan. The cellular composition of these structures was unable to be determined in the current study. A previous study observed epiretinal proliferation at the vitreoretinal interface in RVO eyes [29]. Meanwhile, other studies showed that immunity and inflammation in the retina were involved in complications secondary to RVO, such as ERM and retinal atrophy [3,21,30,31,32]. However, we excluded RVO eyes with ERM in this study in order to reduce the impact on images and MLC count. More follow-up studies are needed to study the outcome of these cells and their relationship to secondary pathological changes.

Another interesting discovery of the study is that the density and distribution of MLCs was affected by retinal edema. The MLC density of the edematous region was less in the acute eyes. Furthermore, we also found a negative correlation between the MLC number and CFT in the acute RVO eyes. The situation is far different in the chronic eyes. Although in the chronic eyes, fewer MLCs were found in the edematous region, this was not statistically significant compared with the density of MLCs in the whole image. Moreover, the correlation between the MLC number and the CFT and MRT was positive in the chronic RVO eyes. Importantly, macular edema secondary to RVO is one of the leading causes of vision loss [33]. The mechanism of macular edema is complex and still unclear [34,35]. Many studies have demonstrated that the breakdown of the blood retinal barrier and retinal vascular hyperpermeability are important in the pathophysiologic process of macular edema associated with RVO [8,28]. Microglia also contributes to the maintenance of the inner blood retinal barrier [36]. Previous research has demonstrated that both the activation of microglia and the invasion of macrophages from the systemic circulation are involved in macular edema [6]. Although the mechanism behind this finding is unknown, we speculated that in our study, the failure of recruitment was responsible for the reduced number of MLCs in the edema region, where the vasculature obstruction and circulatory disturbance occurred during the acute stage. Meanwhile, the restoration of blood circulation in the chronic stage resulted in the activation and recruitment of more microglia and macrophages in eyes with macular edema. A similar theory was proposed in DR, experimentally and clinically [37,38]. Based on the current study, we assumed the same pathological process was present in RVO. Follow-up studies are needed to further confirm this inference through observing the change in MLCs throughout the course of the entire disease and the response of MLCs to macular edema remission. Experimental studies are also required to explain the mechanism involved and to determine the function of these cells in retinal edema.

In the current study, to minimize the effects of treatment, we only included treatment-naïve individuals in the acute group. However, most people in the chronic groups had received different treatments. Due to the treatment effect, a negative result when comparing the acute with chronic groups does not illustrate that there was no relationship between cell numbers and the course of the disease. We also found that there was no significance in MLC density and retina thickness whether comparing the treated group with the untreated group or comparing among subgroups with different treatment methods. Considering the inconsistent time interval between observation and treatment in the retrospective study, the current study cannot draw definite conclusions about the relationship between MLCs and treatment. Further prospective studies should be conducted to investigate the MLCs response to various treatment modalities and the significance of MLC instructional therapy.

The present study did have some limitations. First, it is a retrospective cross-sectional study. Perspective studies are needed to illustrate the change in MLCs during the whole course of the disease. Second, because of the limitation of clinical OCT, MLC morphological changes in RVO can only be described, not quantified. In addition, because of the imaging technology, it was difficult to use blinding processing of the images. Although only a few images in the study were corrected manually during the quantification of MLCs, no blinding used may have some impact on the result. Another limitation is that after excluding eight eyes with other diseases, only sixty-two fellow eyes were included in our study; fellow eye may be a selection bias. Moreover, due to the lack of data on electroretinography and best-corrected visual acuity in some patients, we did not divide patients into ischemic RVO and non- ischemic RVO or compare MLC density between them. In addition, although we compared differences between chronic RVO patients with different treatments, the number of cases in each subgroup were insufficient and observation points could not be unified. No firm conclusions could be drawn about the relationship between treatment and MLC density. In addition, because of incomplete data, we did not consider the influence of system factors such as hypertension, hyperlipidemia, rheumatoid, oral intake medications, and smoking status, etc., which may have a potential impact on the outcome. Lastly, clinical observation is not enough to explain the origin and function of these cells. Further experimental studies are needed to explore the mechanism of this phenomenon. 

In general, the significantly increasing numbers of MLCs on ILM were found in both acute and chronic RVO eyes. We also found that MLC density was relative to retinal edema. The current study provides an insight from the clinic that MLCs play a role in the inflammatory response and retinal edema, secondary to RVO. The investigation of MLCs is helpful for understanding their pathophysiology and for finding therapeutic targets for RVO complications. Last but not least, the observation of MLCs in clinical settings is a simple and non-invasive method to evaluate the severity of the inflammatory response. As an imaging biomarker, MLCs might contribute to estimating the prognosis of RVO and to guiding treatment in the clinic.

## Figures and Tables

**Table 1:** Demographic characteristics of subjects.

	RVO Eye					Control Eyes	*p* *
	Acute RVO Eyes		Chronic RVO Eyes		All RVO Eyes		
	CRVO Eyes	BRVO Eyes	CRVO Eyes	BRVO Eyes			-
Subjects, n	15	28	13	16	72	64	-
Sex (female), n (%)	2 (13.33)	15 (53.57)	6 (46.15)	7 (43.75)	30 (42.86)	28 (43.8)	*p* * = 0.33
Age, mean ± SD	53.73 ± 8.23	57.50 ± 11.23	51.00 ± 14.58	56.06 ± 14.10	55.2 ± 12.0	54.39 ± 12.01	*p* * = 0.69
BCVA (logMAR)	1.27 ± 0.36	0.81 ± 0.41	0.50 ± 0.27	0.32 ± 0.30	0.68 ± 0.45	0.22 ± 0.20	*p* * < 0.001
Missing, n	3	6	1	1	11	11	-
Refractive error (D), mean ± SD	−0.35 ± 1.70	−1.14 ± 1.17	0.17 ± 1.21	0.61 ± 0.87	0.23 ± 1.39	0.19 ± 1.16	*p* * = 0.78
Missing, n	6	13	5	6	30	30	-

*: Comparison between all RVO eyes and control eyes.

**Table 2:** OCTA parameters and MLC count in RVO eyes and control eyes.

	RVO Eyes (n = 72)			Control Eyes (n = 64)	*p*
	Acute RVO Eyes (n = 43)	Chronic RVO Eyes (n = 29)	All RVO Eyes		
Disaese duration (days)	33.00 (43)	265.00 (535)	65.50 (177)	-	*p* ^#^ < 0.001
MRT (μm)	422.40 (126.69)	340.82 (112.48)	388.05 (140.21)	291.52 (16.56)	*p* * ^#^ < 0.001
CFT (μm)	555.31 (382.93)	276.65 (492.09)	502.16 (469.42)	207.84 (18.49)	*p* * < 0.001 *p* ^#^ < 0.01
VD of DCP (%)	38.82 ± 11.14	43.90 ± 9.45	40.87 ± 10.73	56.60 ± 3.88	*p* * < 0.001 *p* ^#^ < 0.05
VD of SCP (%)	55.76 ± 6.90	50.23 ± 8.12	53.54 ± 7.85	51.48 ± 5.08	*p* * ^#^ < 0.05
MLC count	388.00 (353)	422.00 (393)	391.00 (352)	220.50 (220)	*p* * < 0.001 *p* ^#^ = 0.22
MLC density (cells/mm^2^)	11.53 (10.37)	12.53 (11.66)	11.61 (10.44)	6.55 (6.53)	*p* * < 0.001 *p* ^#^ = 0.22

MRT—mean retina thickness; CFT—central fovea thickness; VD—vessel density; DCP—deep capillary complex; SCP—superficial capillary complex; MLCs—macrophage-like cells. *—comparison between all RVO eyes and control eyes. #—comparison between acute group and chronic group.

**Table 3:** Comparison of MLC parameters between retinal edema regions and non-edematous regions in RVO eyes.

	Edematous Region (n = 63)	Non-Edematous Region (n = 63)	Whole Image	*p* ^a^
Area (mm^2^)	9.14 (13.07)	24.52 (13.07)	33.66	-
MLC count (cells)	62 (165)	279 (268)	394 (369)	*p* * < 0.001 *p* ^#^ = 0.001
MLC density (cells/mm^2^)	7.91 (10.97)	13.85 (12.21)	11.70 (10.96)	*p* * < 0.001 *p* ^#^ < 0.01

MLCs—macrophage-like cells. Definition of edematous region was described in Materials and Method. a—Friedman test used for comparison; *p* has been adjusted by Bonferroni correction for a number of tests. *—comparison between edema region and non-edematous region. #—comparison between edema region and whole image.

## Data Availability

The data from this study are available from the corresponding author upon request.
